# Quantifying the Geometric Shell Shape between Populations of True Limpets *Lottia Mesoleuca* (Mollusca: Lottidae) in Colombia

**DOI:** 10.3390/ani10040675

**Published:** 2020-04-13

**Authors:** Ana María Echeverry, Edgardo Londoño-Cruz, Hugo A. Benítez

**Affiliations:** 1 Facultad de Ciencias Agropecuarias, Universidad Nacional de Colombia, Sede Palmira 763531, Colombia; amecheverryo@unal.edu.co; 2 Departamento de Biología, Universidad del Valle, Cali 760032, Colombia; edgardo.londono@correounivalle.edu.co; 3 Centro de Investigación de Estudios Avanzados del Maule, Universidad Católica del Maule, Talca 3466706, Chile

**Keywords:** geometric morphometrics, shape variation, shell shape

## Abstract

**Simple Summary:**

The family Lottidae in the Colombian Pacific is made up by a number of species with diverse morphologies that are products of their specific habitats. Several tools for studying morphological diversity have been used to identify species groups, among them traditional morphometrics and lately geometric morphometrics. Different populations of the true limpet species *Lottia mesoleuca* were examined to determine their population variation in Colombia, evaluating aspects of geometric variation in their shells. The results achieved indicate that geometric morphometrics has enough potential to identify small differences between populations of the true limpets independently of their similar morphologies.

**Abstract:**

The increasing activity in morphological studies has provided new tools to analyses the shape quantitatively, these quantitative measurements allow the researcher to examine the variation in shape and perform analysis to examine the quantitative differences among the species shapes, where geometric morphometrics has rendered great results in the last years. This study was focused on assessing the morphometric variation between populations of *Lottia mesoleuca* of the family Lottidae, an abundant group of gastropods in the rocky ecosystems of Bahía Málaga and Isla Gorgona (Colombian Pacific). This family has a high morphological diversity, making the identification of some morphotypes problematic work. Geometric morphometrics methods were applied on the shell using dorsal, lateral and ventral views. Different multivariate analyses were performed to differentiate the groups of species and populations (principal component analysis, morphological distances comparisons and grouping analysis by means of the Ward method). The results indicate that individuals of the species *Lottia mesoleuca* have key geometric characteristics associated to the different populations (depth intertidal zones) for classification, being the geometric shape of the shell enough to determine morphotypes between the different populations studied. Aspects associated with the combination of ecological variables with morphometric ones are necessary to be able to visualize with a higher resolution the structural complexity of populations and their adaptation processes. Furthermore, it is obvious that there is a strong need to conduct more explorations of environmental and ecological processes that provide some insight on why the morphological characteristics are so variable in the same species.

## 1. Introduction

One of the most outstanding and remarkable aspects of nature is morphological variability, with one of the main challenges lying in compared anatomy, where describing and quantifying morphological variation is essential to identify and classify organisms, analyze the structure–function relationship, understand ecological and behavioral aspects, recognize adaptations and rebuild macroevolutionary patterns. So, in the first approaches to morphometric studies, traditional morphometrics combines linear measures between anatomic points with single- and multivariate statistical techniques to analyze differences in size and shape [1].

Although traditional morphometrics has been used and has had good results, it does not take into consideration the direction of morphological differences, which in turn restrict the visual reconstruction of conformation. Therefore, one of the great advances in geometric morphometrics, compared to traditional morphometrics (based on lengths, ratios or angles) is that it captures shape geometry and allows its inclusion in the analyses, keeping the relative position of the morphological reference points and allowing its visualization at all times from any operation performed. But what is most important in geometric morphometrics is the shape described by landmarks [2,3,4]. Homologous structures (similar origin and location within the anatomy of the specimen) whether for their phylogenetic, structural, functional or developmental meaning [2,5,6], are those deriving from a common ancestral structure [1]. According to this definition of homology, “landmarks are required to be discrete, that is, that they are clearly distinguishable from the structures around them, allowing in this way that any construing of the variation observed can have a biological sense” [5,7].

Colombia is considered one of the two countries in the world with the highest biodiversity, both in terms of species and ecosystem richness. Marine biodiversity can be translated economically or ecologically, since it is a source of food and biotechnological resources, as well as indicators of the environmental quality and functioning of ecosystems (trophic networks). The main threats to marine biodiversity include overexploitation, habitat degradation, pollution, global warming, biological invasions, and other anthropogenic stressors, most of them in coastal areas. Given the above, it is currently vitally important to know the species present in ecosystems to understand and conserve biodiversity [8,9].

Mollusks represent the second richest phylum of marine organisms, with more than 50,000 described species [9]. Since the coastal and insular areas of Colombia are surrounded by two oceans, Colombia has hosted one of the richest species-rich malacofaunas in America [10,11]. However, few taxonomic studies have been conducted on mollusks from Colombia, and most have focused on the Pacific coast.

The order Patellogastropoda, inhabits all the world’s oceans, from tropical to polar regions and from the depths of the sea to well above the high tide line. Therefore, it is a diverse and ecologically important group [12,13]. The correct identification of the order at species level has been complex particularly because of components associated with phenotypic plasticity and variability of the shell characters (most commonly used characters for their identification, such as color, texture, patterns, spirals, scars, ribs) as well as length, width, height and apex position. Additionally, this “high morphological variability of the shell of these organisms has given rise to a huge confusion in their taxonomy” [14,15,16]. Historically, taxonomic studies of the Lottiidae family have been based on the morphology of the shell and on the characters of the radula [17,18,19,20], but the high plasticity of the shell has led to taxonomic confusion and a failure to recognize complex species [15,21].

Studies in the classification of these organism have identified that different species of limpets prefer different intertidal depth zones; species from the Genus *Lottia*, particularly *L. mesoleuca* can be easily found in the low and medium zone, by contrast with other genera like *Scurria* with a broader distribution, living in the high-, medium- and low-depth zones. Researchers have suggested that shell morphology in limpets is correlated with the depth gradient, where limpets in lower depths tend to have higher shells. This depth gradient is opposite to previous studies that have reported a morphological distinction between intertidal and subtidal morphotypes [19,22,23,24]. On the other hand, the muscular effort to stay adhered to the rock in environments with high energy, may lead to a distortion in the mantle, resulting in higher shells [25,26,27]. Therefore, the shell morphology may change in response to the degree of exposure to swell in many coasts [28,29,30,31]. Other factors of environmental stress associated with physical factors, such as temperature, desiccation, and salinity, among others, may also cause variations in shell shape.

Laboratory experiments Lindberg and Pearse [32] have confirmed that environmental conditions, topography or substrate and food, give rise to changes in color and morphology of *Lottia asmi* and *L. digitalis* limpets.

To quantify the potential variation in shell shape, geometric morphometrics has given some classification tools by means of landmarks and curves where the geometry of the shell and its specific variations in each homologous point have been useful for researches, enabling them to draw better conclusions about the aspects of morphological changes in taxonomically problematic groups [33,34]. In this manner, with the landmarks and semi-landmarks it has been possible to provide further anatomic information for structures defined by curves or surfaces, which cannot be delimited by homologous marks, and hence, which can be included in the shape analysis [35,36].

Based on the above, this article intends to evaluate if geometric morphometrics tool can provide a better understanding of morphological variation between the populations across Colombia in *Lottia mesoleuca*, mainly associated with Bahía Málaga (Los Negritos, Isla Palma and Morro Chiquito) and Isla Gorgona (La Ventana and El Muelle), with the final purpose of proposing this tool as a reliable alternative for better morphological identifications and population differentiation using the geometric shape of organisms.

## 2. Materials and Methods

### 2.1. Sampling and Acquisition of Data

**Figure 1:**
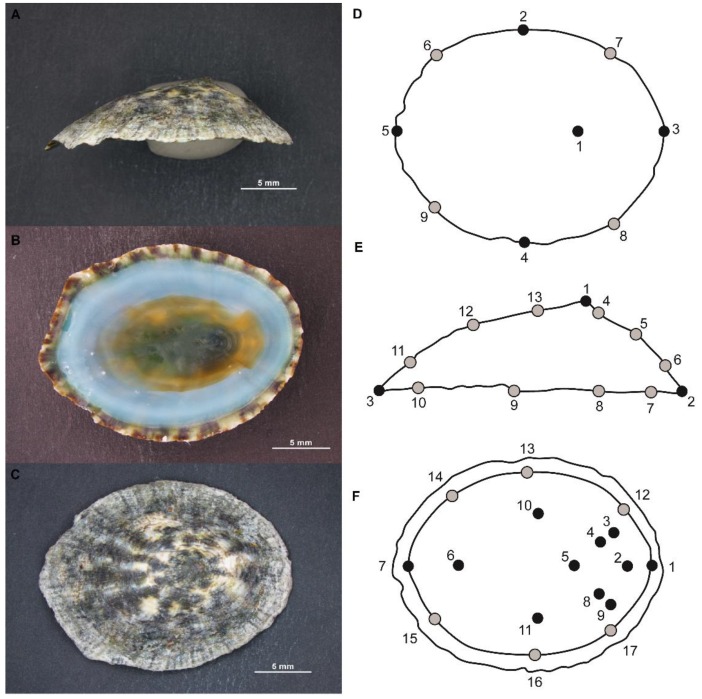
*Lottia mesoleuca* (Menke, 1851) representation in A: lateral, B: vental and C dorsal view and their schematic diagram with the representation of the distribution of landmarks in the three positions (**A**,**D**)—Dorsal (9 landmarks), (**B**,**E**)—Lateral (13 landmarks) and (**C**,**F**)—Ventral (17 landmarks).

Photographs of four population of *Lottia mesoleuca* were taken with a Nikon SMZ1500 stereoscope of dorsal, lateral and ventral planes (Figure 1A–C), for this procedure the individuals were fixed in a mold using plasticine, so that they kept a stable shape all the time and they were photographed with the corresponding scale; from those, 48 individuals corresponded to Isla Gorgona, 116 from Isla Palma, 11 from Los Negritos and 38 from Morrochiquto. To identify the organisms, a first approach was implemented separating the individuals according to their morphological characters (foot color, tentacle color, shell characters). Then, a final separation was conducted into different shell morphotypes, considering the apex position, the profile, shell aperture, type of inclination, shell sculpture and type of margin, and the internal features of the shell, using the corresponding taxonomic keys [37] and a revision of the radulae to complement the individual characteristics.

### 2.2. Morphometrics and Multivariate Analyses

Three views of the shell were digitized using 9 dorsal, 13 lateral and 17 ventral landmarks (black) and semilandmarks (grey) in the images using the software program tpsDig2 [38] (Figure 1D–F).

To analyze the information of shape, the effects from position, orientation and scale are removed by a generalized Procrustes superimposition analysis [1,39,40]. In order to see the differences or similarities in body shape of the species, a principal component analysis (PCA) was conducted in the program R 3.5.0 using the package geomorph [41,42]. This is one of the simplest multivariate techniques intended to synthesize information reducing the dimensionality of variables, so that the least information possible is lost and data variation can be described properly [43,44].

To establish if there were differences between the means of the population shapes, the Procrustes distances between groups were used extracted from a canonical variate analysis [45] performed in the software MorphoJ 1.06d [46]. The significance of pairwise comparisons is established by means of Bonferroni’s adjustment, which is calculated with the likelihood of getting a significant result equivalent to 95%, in the number of possible combinations [47].

Once the pairwise comparisons were completed, grouping according to Ward’s method was performed, calculating distances as similarity measures between the objects, in order to set up groups [48]. The objects or groups are joined under the criterion to make them the most compact and homogeneous possible. Heterogeneity is evaluated as the sum of distance squares of each object in the group considered to be at the center of such group [48]. The criterion is to join those objects for which this value is minimum. Although final representations differ, in the distances from some objects to others, the groups so found are normally the same.

## 3. Results

### 3.1. Morphological Component

#### *Lottia Mesoleuca* (Menke, 1851)

The features of the *L. mesoleuca* shells corresponds to conical shell with oval aperture. With a medium profile, subcentral apex in almost all the specimens well marked, the shell may be smooth or with radial innervations. External color is variable (green, dark and light brown, grey and beige), in the inner side, color can go from greenish blue, whitish and some of them are tanned, some specimens have white lines going up to the shell edge, and others have large areas with white specks or freckles or mesh pattern. The shell may, in some cases, be eroded. The mantle, in some cases, may have pigmentation. Dimensions of specimens found: Length: 5 to 34 mm, width: 2 to 28 mm, height: 5 to 8 mm and their habitat are rocky intertidal. 

The radula of *L. mesoleuca* correspond to a docoglossan radula, which is characteristic of this family. It has long membrane on which radular teeth are arranged in transverse rows, absent a central tooth in each row, 3 pairs of lateral teeth (DL).

### 3.2. Morphological Variation

The PCA included all the individuals to assess their general variation of shape between the populations of *L. mesoleuca*. Variability at a general level in the dorsal view is represented by the shell width and length, measured from the most anterior and posterior area of the apex. The first principal component (PC1) accounted for 34.8% of the variance and PC2, 14.8%. In the lateral view, the first principal component (PC1) accounts for 51.7% and PC2, 16.3% of the variance; although some points are still overlapped it is possible to identify a clearly separation between specimens from Isla Gorgona represented, showing that is fairly irregular with the apex somewhat depressed inwards. The PCA of the ventral view shows that the internal traits of the shell PC1 accounted for 28.07%, PC2 23.4% of the ventral shape variance, in this view was possible to observe how the muscular scar is smaller and shorter in the population with less exposure to waves, compared to a fairly long and wide one, respectively, nonetheless the variation is lower in comparison to the other views. 

Statistically significant differences were found comparing the morphological distances between population using the Procrustes distances between the three views of the body shape of *L. mesoleuca* (Table 1), for these geographical variations, it is important indicate that in Isla Palma there is a bug abundance of individuals and the population Los Negritos did not differ very much in shape for the lateral and ventral view. After running Bonferroni adjustment (α = 0.008), and based on the matrix of significant distances between each pair of species, statistically significant shape differences were found between *L. mesoleuca* different localities (Table 1).

**Figure 2:**
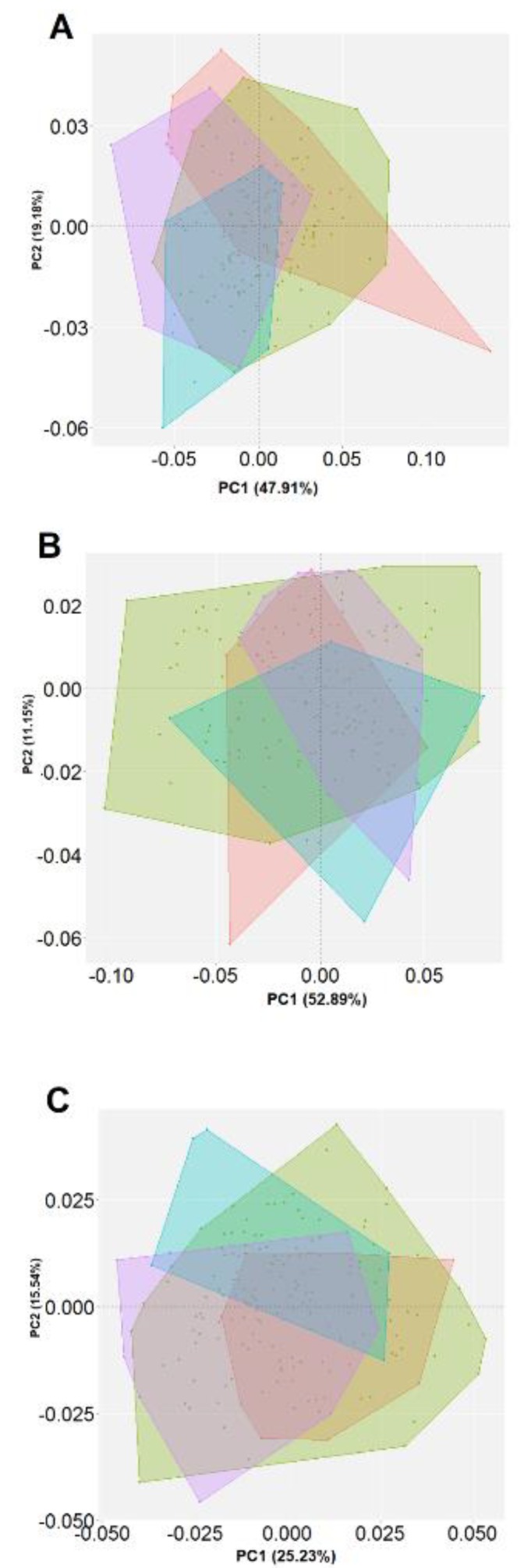
Principal component analysis (PCA) comparing the (**A**) dorsal, (**B**) lateral and (**C**) ventral view of *Lottia mesoleuca*. The figures show different colored confident ellipses (95%) for the geographical location red: Isla Gorgona, green: Isla Palma, ligh blue: Los Negritos, purple: Morro Chiquitito.

In the dorsal view it is observed to have the greatest variation in length from the apex to the posterior part, and around the contour in the widest points, they are displaced backwards or forwards. Although no differences are perceived regarding the different PCs between populations, there are significant differences between individuals of Gorgona Island and Málaga Bay, but there are clear differences between Morro Chiquito and Los Negritos (Table 1, Figure 2A).

In the lateral view, variations in maximum and minimum distortions come from height and length, where in the negative end towards the left side, it is caused by a very long and shallow shell, in the lower end, shorter and rather higher and with subcentral apex. In the upper end, from the apex to the posterior end, it is more convex, and at the right end the shell is sturdier. Differences were observed between Gorgona Island and Palma Island and Morro Chiquito, and between Los Negritos and Palma Island (Table 1, Figure 2B).

**Figure 3:**
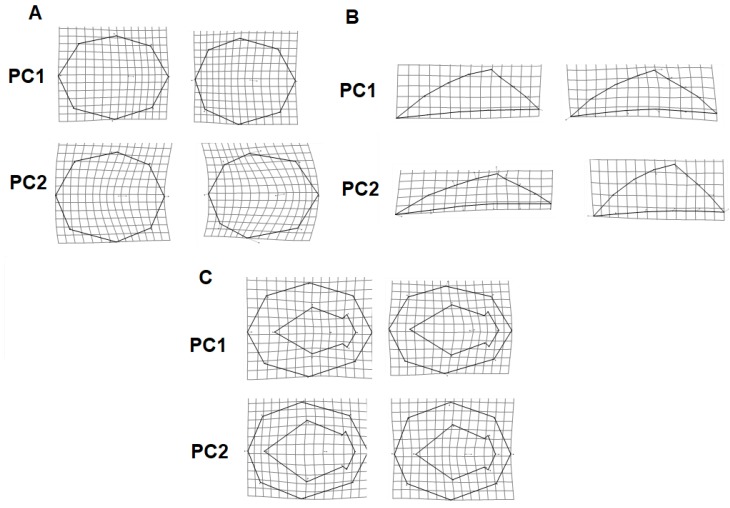
Wireframe visualization of the maximum and minimum shape of the principal component 1 and 2, comparing the (**A**) dorsal, (**B**) lateral, and (**C**) ventral view of *Lottia mesoleuca* species and their respectively negative (left) and positive (right) wireframe visualization of the average shape for geographical shape variation.

For the ventral vew, the shape variation of most individuals of Los Negritos zone are explained in the positive zone of PC2, where the muscular scar and the intermediate zone keep their own shape characteristics, so that in the minimum distortions they are shorter and in the maximum they are longer. There are significant differences among all the individuals from all the locations (Table 1, Figure 2C). An average shape variation for the multiple views was calculated for the minimum and maximun axes of the PCA where geographical shape variation can be distinguished between groups (Figure 3).

**Figure 4:**
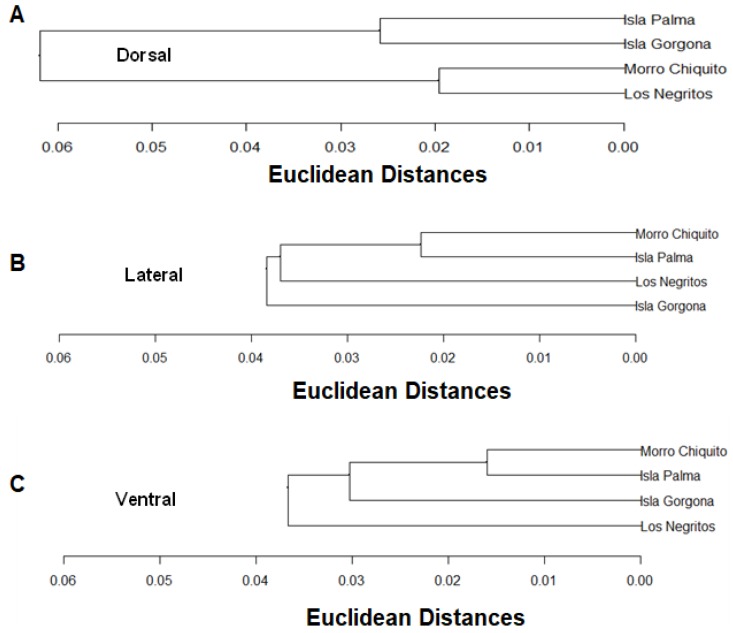
Dendogram of similarity of the Euclidean distances in the views ((**A**): Dorsal, (**B**): Lateral and (**C**): Ventral) in Málaga Bay and Gorgona Island.

Once the Euclidian distance matrices for similarity have been completed, the analysis for grouping by the Ward method was conducted, which is more discriminatory, in order to determine the grouping levels. A grouping for all the views was performed (Figure 4). Similarities in the dorsal view are more between individuals of Palma Island and Gorgona Island, and between those of Morro Chiquito and Los Negritos. The lateral view shows similarity between individuals from Morro Chiquito and Palma Island, followed by Los Negritos and Gorgona Island, which have similar features in the type of rock. The ventral view is perhaps the most variable in shape, which may be caused by differences in the type of rock that each population predominantly lives upon. Variation in rock type may be associated with ontogenetic variation [49,50,51], showing, therefore, that similarities are in the individuals from Morro Chiquito and Palma Island, followed by those from Gorgona Island and the least similar ones in Los Negritos.

## 4. Discussion

The current study shows the importance of geometric morphometrics tools to study shape disparity correlated to environmental factors [3,52,53]. The shape variation in *Lottia mesoleuca* have shown diverse strategies to inhabit in different depth zones across the intertidal populations, these strategies may be related with patterns of morphological adaptation among their distribution sites, which may be related with the environmental pressure across the intertidal. Specimens whose traits present more plasticity particularly shell shape in response to the environment, have been extensively identified as having characteristics of successful invaders by contrast with species with a lower pattern of plasticity [28,54,55,56]. Morphometric traits, in some cases, pose challenges when being used to identify species, since they are influenced by both environmental and genetic factors [53,57]. *L. mesoleuca* in Málaga Bay and Gorgona Island have a wide morphologic variability, such as different texture, relief, height and position of the apex, as well as shell width and length and even multiple color patterns and variety on the shell, which are considered to be adaptive characteristics modelled by environmental conditions and, in some cases, a response to predation [58,59], the primary source of food [60,61] and even due to genetic factors [62,63]. 

It has been suggested that soft tissue characters are too conserved to be used as a character to differentiate a species; on the other hands, the shells with their characteristics tend to be variable enough to solve phylogenetic relationships [64]. However, since the limpets of the family Lottiidae have simple shells without easily identifiable morphological traits, the problem becomes worse for the plasticity of the shell morphology [65]. This is the reason why studies have been conducted to identify species in other marine organisms, including some limpets, such as the case of *Cellana strigilis*, which has phenotypic plasticity [66,67,68]. Such phenotypic plasticity may be due to the natural selection during evolution or during the life cycle, the environment, predation [30,69,70,71,72,73] or by means of ontogenetic changes [74,75,76] associated with environmental conditions, such as temperature [28,77,78].

In future studies, it would be important to consider other morphological traits besides the shell, such as soft tissues: the edge of the exterior mantle that is coated with a row of small sensory tentacles, which may have different characteristics between species, although they might be not so notorious in plain sight. These edges, in some cases, may or may not have color. Additional features may also be considered, like the shell thickness that provides information about swell intensity, since this would allow identifying the compressive strength and, hence, resistance to predation; for example, thin shells may are more exposed and susceptible to predation [79]. Predation comes from a great variety of predators: adult crabs, birds, fish and sea snails among them. But in reality, true limpet morphology and distribution probably result from a complex and diverse set of interactions between predation, competence and environmental stress factors. Limpets located in the higher intertidal zone show mortality not only due to some predators, but mainly associated with physical factors (temperature, desiccation, salinity and others), whereas mortality in the lower intertidal zone is predominantly as a result of predation and other biotic interactions [80]. Therefore, this research allowed us to determine that geometric morphometrics tools facilitated the classification and identification of the shape variation between *Lottia mesoleuca* populations and the combination with new multivariate approaches allowed us to suggest that the observed shape–environment association could be a result of the high plasticity of this species regarding their environment, such that in the future a more powerful combination with ecological traits using partial least squares [81,82,83,84] analysis is needed.

## Figures and Tables

**Table 1:** Results of the Canonical Variate analysis with Procrustes distances and the respective *p* values, for the respective views and populations of *Lottia mesoleuca*. Statistical significant values are in bold.

Population	Isla Gorgona (Proc. *p* Value)	Isla Palma (Proc. *p* Value)	Los Negritos (Proc. *p* Value)
Lateral			
Isla Palma	0.0324 (**<0.0001**)		
Los Negritos	0.0199 (0.5609)	0.0232 (0.1318)	
Morrochiquito	0.0364 (**<0.0001**)	0.0178 (**0.0340**)	0.0263 (0.0929)
Dorsal			
Isla Palma	0.0412 (**<0.0001**)		
Los Negritos	0.0689 (**0.0001**)	0.0407 (**0.0001**)	
Morrochiquito	0.0539 (**<0.0001**)	0.0309 (**<0.0001**)	0.0226 (0.2673)
Ventral			
Isla Palma	0.0149 (**0.0006**)		
Los Negritos	0.0228 (0.0851)	0.0165 (0.1102)	
Morrochiquito	0.0226 (**0.0004**)	0.0128 (**0.0087**)	0.0252 (**0.0027**)
